# Geological Hydrogen Storage: Geochemical Reactivity of Hydrogen with Sandstone Reservoirs

**DOI:** 10.1021/acsenergylett.2c01024

**Published:** 2022-06-03

**Authors:** Aliakbar Hassanpouryouzband, Kate Adie, Trystan Cowen, Eike M. Thaysen, Niklas Heinemann, Ian B. Butler, Mark Wilkinson, Katriona Edlmann

**Affiliations:** † School of Geosciences, University of Edinburgh, Grant Institute, West Main Road, Edinburgh EH9 3FE, U.K.

## Abstract

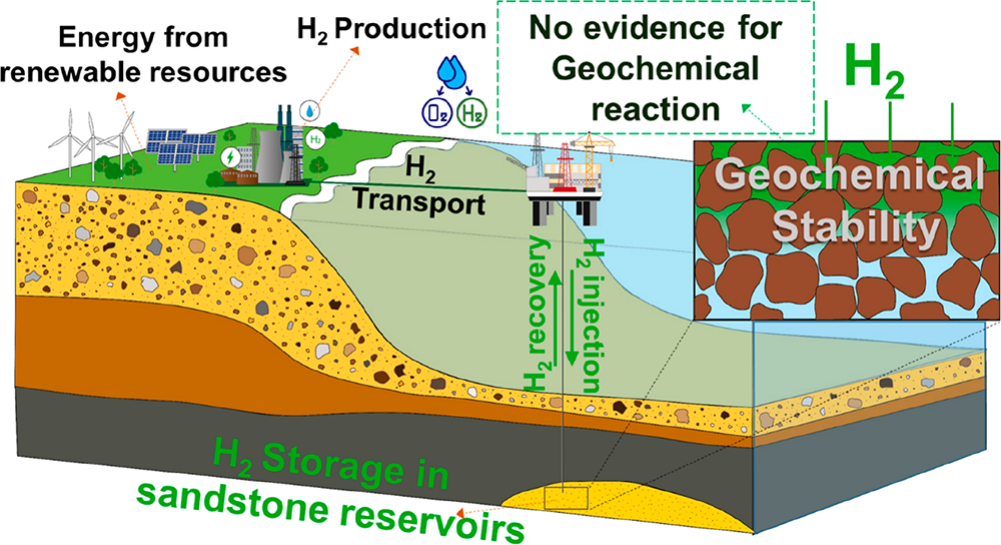

The geological storage of hydrogen is necessary to enable the successful transition to a hydrogen economy and achieve net-zero emissions targets. Comprehensive investigations must be undertaken for each storage site to ensure their long-term suitability and functionality. As such, the systematic infrastructure and potential risks of large-scale hydrogen storage must be established. Herein, we conducted over 250 batch reaction experiments with different types of reservoir sandstones under conditions representative of the subsurface, reflecting expected time scales for geological hydrogen storage, to investigate potential reactions involving hydrogen. Each hydrogen experiment was paired with a hydrogen-free control under otherwise identical conditions to ensure that any observed reactions were due to the presence of hydrogen. The results conclusively reveal that there is no risk of hydrogen loss or reservoir integrity degradation due to abiotic geochemical reactions in sandstone reservoirs.

To combat the adverse effects of climate change, legislation, emissions objectives, and climate protection agreements have been established. This has instigated a drive toward the advancement and widespread uptake of low-carbon technologies and resulted in a significant increase in renewable energy production and consumption in recent years.^1^ One of the main challenges associated with renewable energy resources is their inherent variability; wind turbines and solar panels are vulnerable to natural fluctuations in wind strength, direction, and available sunlight hours. This variability does not align with the supply and demand requirements of the human population and increases the need for large-scale energy storage technologies to support the increasing growth in low-carbon renewable energy. Hydrogen generated from the electrolysis of water powered by excess or dedicated renewables has been identified as a low-carbon energy vector that can provide the necessary energy storage to support renewables and provide flexible supply and demand balancing at hourly, daily, and interseasonal time scales. Hydrogen also provides the potential to decarbonize “hard to abate” sectors such as heavy industry and heavy-duty vehicles, railways, and shipping.^2^

The hydrogen-to-carbon ratio in hydrocarbon fuels determines their energy density because hydrogen (141.86 MJ·kg^–1^ energy density) has the highest energy density among hydrogen-based fuels (e.g., 55.5 MJ·kg^–1^ for CH_4_). However, hydrogen is a very low-density gas (0.084 kg·m^–3^, compared with 0.668 kg·m^–3^ for methane, at 20 °C and 1 atm), and while surface storage solutions are well-developed, they are limited by storage and discharge capacities. With a density of 70.8 kg·m^–3^, liquid hydrogen would still not be a practical choice for interseasonal energy storage because of limited above-ground energy storage capacities. As an example, the North Sea Leman gas field in the U.K. can store the same amount of energy as 3 × 10^8^ m^3^ of liquid H_2_, which would require nearly 4000 football pitch sized tanks each 10 m in height.^3^ Yet, liquid hydrogen has a continuous boil of around 0.4% per day for a storage volume of 50 m^3^, which reduces its efficiency and is therefore inefficient for long-term use. Delivering the storage and discharge capacities required for interseasonal energy storage (gigawatt hour (GWh) to terawatt hour (TWh)) will require geological storage in suitable formations such as salt caverns (GWh), saline aquifers, and depleted hydrocarbon reservoirs (TWh). The largest storage and discharge capacities are provided by porous geological formations, including depleted gas fields, which feature a porous and permeable reservoir formation, a caprock, and a trap structure.^3^ Injected hydrogen displaces the formation fluids with vertical migration prevented by the impermeable caprock and lateral migration prevented by the 3D trapping structure, allowing the stored gas to be injected, stored, and recovered.

Geological hydrogen storage is considered a viable option to ensure reliable energy supplies, with over 1000 TWh of natural gas energy storage capacity in porous rocks currently in operation around the world. The establishment of geological hydrogen storage sites will balance seasonal fluctuations in renewable energy generation and ensure consumer supply is met by producing and storing hydrogen during periods of off-peak demand and producing during periods of increased demand. Geological hydrogen storage can also enable further penetration of renewable energy sources within the energy system to support the global net-zero ambitions. Experience with porous geological hydrogen storage was developed during the storage of town gas (containing ∼50% H_2_, with CH_4_, CO_2_, CO, and N_2_^4,5^), where town gas storage sites in Germany, France, and the Czech Republic were successfully operated for decades before they were converted to natural gas storage in the 1980s. Additional experience of geological gas storage in porous rocks has been gained through the commercial operation of over 670 natural gas storage sites and over 30 carbon dioxide storage sites.^6−8^ Published studies consider geological hydrogen storage to be technically feasible; however, several reviews have identified challenges which must be addressed to prove the safe containment and necessary recovery efficiencies of hydrogen in porous reservoirs.^3,9−13^ The major barriers currently restricting the development of hydrogen storage are the following: (i) Hydrogen is characterized by a lower viscosity and higher mobility than natural gas and carbon dioxide.^14,15^ Therefore, the behavior of injected hydrogen should be investigated in terms of mobility and multiphase properties to ensure recovery efficiencies are maintained.^16,18^ (ii) Hydrogen acts as an electron donor for a variety of microbial processes. The occurrence and behavior of hydrogenotrophic microbes must be investigated to determine the impact of potential hydrogen consumption losses and compositional changes of the stored hydrogen.^17,19,20^ (iii) The promotion of abiotic geochemical reactions between the reservoir rocks, formation fluids, and stored hydrogen. These reactions may be detrimental to geological hydrogen storage by altering the composition of the stored hydrogen and causing mineral precipitation and dissolution which may impact reservoir integrity and recovery efficiencies.

Experience of town gas storage in Ketzin (Germany) and Beynes (France) provides context to the potential significance of geochemical interactions in underground hydrogen storage. In both cases, alterations to the composition of stored gas were observed. Bourgeois et al. (1979)^21^ suggest that the increased concentration of hydrogen sulfide observed at Beynes can be accounted for by the abiotic reduction of pyrite as opposed to the action of sulfate-reducing bacteria. Reitenbach et al. (2015) suggest that the hydrogen partial pressure (5–10 MPa), temperature (25 °C), and alkalinity that characterize the Beynes storage site support this argument. At Ketzin, gas losses in the order of 2 × 10^8^ m^3^ were observed between 1964 and 1985; the processes causing the gas loss and evolution of gas composition have not been identified but are not considered to be sufficiently explained by microbial degradation alone.^12^ Investigations of abiotic hydrogen reactions in porous media are rare in recent literature and do not sufficiently describe the extent to which geochemical reactions might be expected during geological hydrogen storage. Recent studies into hydrogen geochemical reactivity are restricted to nuclear waste disposal rather than geological hydrogen storage, where storage temperatures, pressures, and fluid saturation conditions are very different.

The injection of hydrogen into subsurface porous reservoirs may promote the transformation of pyrite into iron monosulfide^22^ via coupled dissolution–precipitation and concurrently produce hydrogen sulfide gas, which may not only alter the chemistry of the formation waters and promote further reactions but also lead to the corrosion of subsurface and surface infrastructure.^23,24^ It is important to note that the only observed occurrence of these reactions at likely storage temperatures is in the experiments of Truche et al.^22^ The temperature of the majority of hydrogen storage reservoirs is anticipated to be cooler than the 90 °C used in these experiments. Therefore, further investigation is required to confirm these results.

Increased hydrogen concentrations in porous reservoirs may promote redox reactions, resulting in the oxidation of hydrogen and reduction of electron acceptors (nitrate, Fe^3+^, sulfate, and carbonate);^19^ H_2_-induced redox reactions with iron-bearing minerals such as hematite, and micas and clays containing Fe^3+^ may be observed.^24^ Pervasive dissolution of calcite and anhydrite cements has been observed experimentally upon exposure to hydrogen at elevated temperatures and pressures (10–20 MPa, <40 °C), leading to an increase in porosity.^25^ These results may be significant in the context of the present study, as the pressure and temperature range is anticipated to be similar to that of geological hydrogen storage reservoirs. If proven to occur in a subsurface hydrogen storage setting, the reactions described above hold the potential to alter reservoir and caprock porosity and permeability and thus threaten storage integrity. Therefore, further research is required to investigate the potential impacts of these reactions.

It is possible that under the pressure and temperature ranges and time scales associated with seasonal hydrogen storage, mineral transformations would be kinetically limited.^26^ However, a scarcity of experimental data has resulted in a lack of agreement in recent literature as to the significance of geochemical reactions in porous underground hydrogen storage. An insufficient account of abiotic geochemistry in geological hydrogen storage in the published literature increases uncertainty and means that this remains a technical barrier to the development of geological hydrogen storage. To facilitate the geological storage of hydrogen, the uncertainty associated with hydrogen loss and reduction in reservoir integrity because of abiotic geochemical reactions between the reservoir rocks, formation fluids, and hydrogen must be understood. The research presented in this Letter experimentally recreates subsurface storage conditions to facilitate a representative assessment of the geochemical response of various sandstone samples upon exposure to hydrogen. This work, key in the context of hydrogen storage, addresses an absence of evidence regarding the extent of geochemical reactions in geological hydrogen storage, which is detrimental to the development of the technology.

Herein, we present the results of over 250 batch reaction experiments on a range of different reservoir sandstone samples. H_2_-induced geochemical reactions are identified by comparing element concentrations in solution after H_2_ experiments relative to control experiments. We consider that it is very unlikely that water–rock reactions can occur without a corresponding change in porewater chemistry—the dissolution of existing minerals will increase the concentrations of the associated elements, while the precipitation of a new phase will alter the equilibrium composition of the porewater. The possibility that a volumetrically significant reaction can occur but have no influence on the porewater chemistry is considered to be negligible. The control experiments were conducted at the same temperature, pressure, and fluid salinity but with nitrogen instead of hydrogen (focused on the impact of hydrogen), and bottle experiments were conducted at the same temperature and fluid chemistry but with no added hydrogen or nitrogen and at atmospheric pressure (focused on the impact of pressure). As all the experiments exhibited qualitatively and quantitatively similar results, we focus our analysis on 6 elements associated with potential H_2_-related geochemical reactions associated with acidification of the porewater: pyrite (FeS_2_) reduction or dissolution; gypsum (CaSO_4_·2H_2_O) dissolution; calcite (CaCO_3_) and feldspar (KAlSi_3_O_8_) dissolution. Similar results were obtained for several additional elements, and these are provided in Figure S2 and S3, as well as the Supporting Information data file. Figure 1a–e presents the difference between the concentration in the presence of hydrogen and in the presence of N_2_ (ΔHN) or bottle experiments (ΔHA) to highlight the magnitude of any changes that are specifically a result of hydrogen reactions. All other conditions for each experiment in the hydrogen and control experiments are kept constant, including run time, rock particle size, water-to-rock ratio, temperature, pressure (for N_2_ and H_2_ experiments), salinity, and container type (for N_2_ and H_2_ experiments).

**Figure 1:**
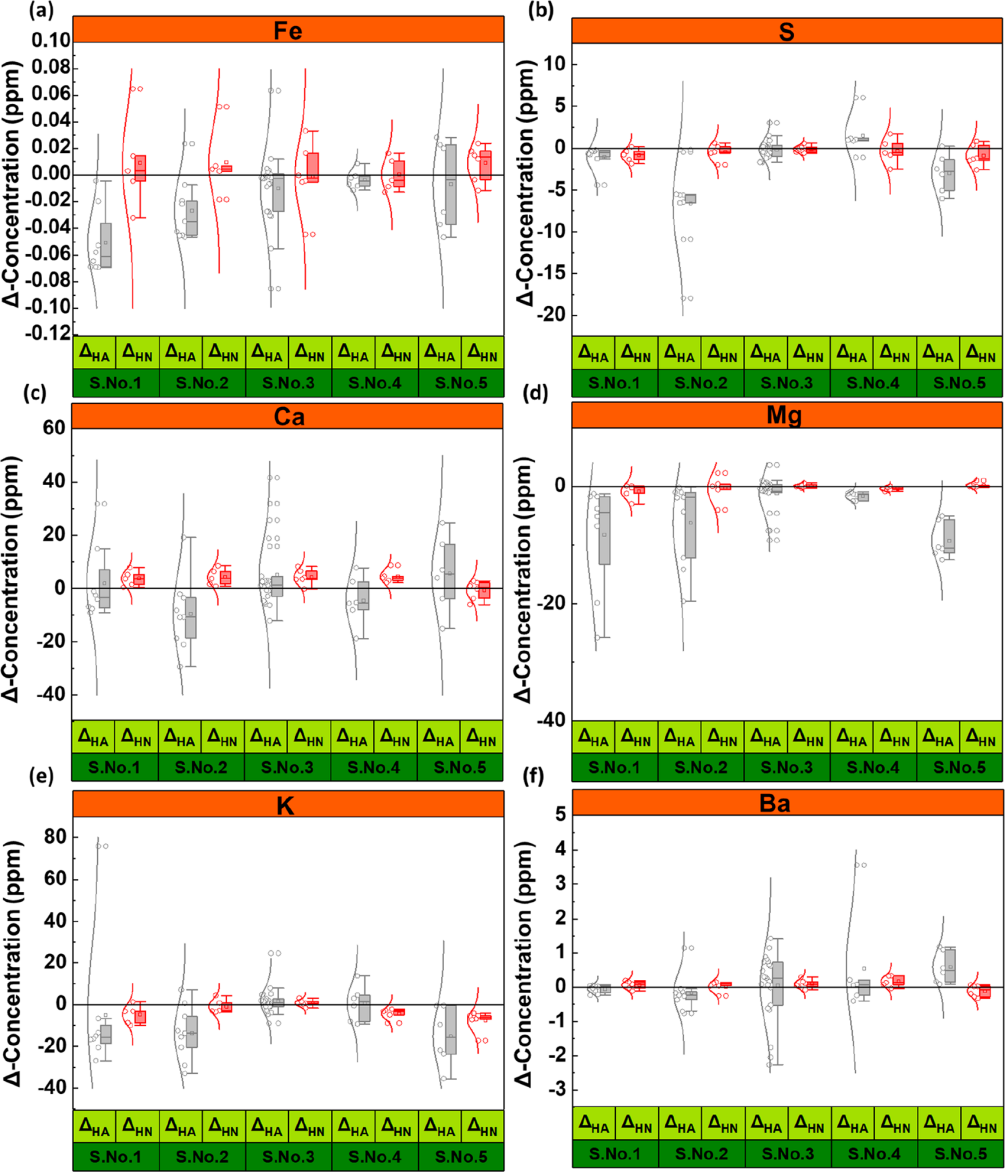
Differences in the concentrations of different elements detected in fluid samples after completing the experiments. Each point belongs to two different experiments at strictly controlled identical conditions with only one changing parameter that is either the presence of H_2_ or N_2_, or the lack of any free gas phase. ΔHN: concentration of the element after experiment with H_2_ deducted from the concentration of the same element after experiments with N_2_. ΔHA: concentration of the element after the experiment with H_2_ deducted from the concentration of the same element after the bottle test. The boxes are determined by the 25th and 75th percentiles, and the whiskers are extended to a maximum of 1.5 × IQR beyond the boxes. The curved lines represent the distribution curves. Each plot contains the following number of data points: (S.No.1-ΔHA: 8), (S.No.1-ΔHN: 5), (S.No.2-ΔHA: 9), (S.No.2-ΔHN: 5), (S.No.3-ΔHA: 23), (S.No.3-ΔHN: 6), (S.No.4-ΔHA: 6), (S.No.4-ΔHN: 5), (S.No.5-ΔHA: 6), (S.No.5-ΔHN: 6). Detailed concentration of the elements can be found in the Supporting Information.

As shown in Figure 1, for ΔHA values, the distribution of the concentration changes is slightly wider, possibly resulting from changes in pressure, which significantly influence the chemical equilibria. For instance, the solubility of calcite and gypsum increases with pressure,^30^ resulting in higher calcium concentrations measured in the higher-pressure experimental runs. Moreover, the experiments were conducted reflecting the time scale of geological hydrogen storage, without external agitation, and as such, this period might not be sufficient to attain chemical equilibria. Accordingly, the pressure of the system could also influence the kinetics of the mineral dissolution and as such affect the approach to chemical equilibrium within the time limit.

Accordingly, ΔHN values are considered to be better representative indicators of whether or not there are any potential geochemical reactions involving H_2_. ΔHN values for all experiments are close to zero for most elements. Nevertheless, for one alkali metal (K) and several alkaline earth metals (Mg, Ca, and Ba), there are some ppm scale fluctuations. To investigate these fluctuations further, 80 experimental runs were performed in 40 pairs to ensure repeatability, with each pair having identical carefully controlled conditions. The differences in concentration of each element are plotted in Figure 2a. Comparing the data presented in Figure 2a with ΔHN values reveals that variations in ΔHN values are within the range of repeatability error. As such the fluctuations observed above can be attributed to minor heterogeneities in the sandstones that create minor differences between the samples tested within the same experiment. The variable rate of mineral dissolution in the water/brine may provide another justification for the fluctuations, as the water–rock system had limited time to react. Moreover, a further set of experiments were run without any rock samples to investigate the potential for impurities within the salt (used to make the brine) or within the reaction vessels to generate fluctuations of elements concentrations. The measured concentration of the elements resulting from these experiments run without any rock phase present is plotted in Figure 2b. As can be seen, impurities in the salt and the dissolution of the glass of the reaction vessel did result in ppm level variations in element concentrations, providing an additional reason for the measured fluctuations in the ΔHN values. In conclusion, the differences in the compositions of experiments with hydrogen compared to nitrogen are negligible and demonstrate an absence of geochemical reactions within the time frame of geological hydrogen storage for any of the sandstones tested within this study. To further verify the validity of this claim, we measured the composition of the reacted gas after the experiments with a mass spectrometer. Water vapor was the only impurity detected up to ppb levels (see Figure S4), indicating that hydrogen can be recovered for end-use consumption. Moreover, the logged temperature and pressure of the experimental system did not change during the experiments, which is further evidence for this conclusion (see Figure S5).

**Figure 2:**
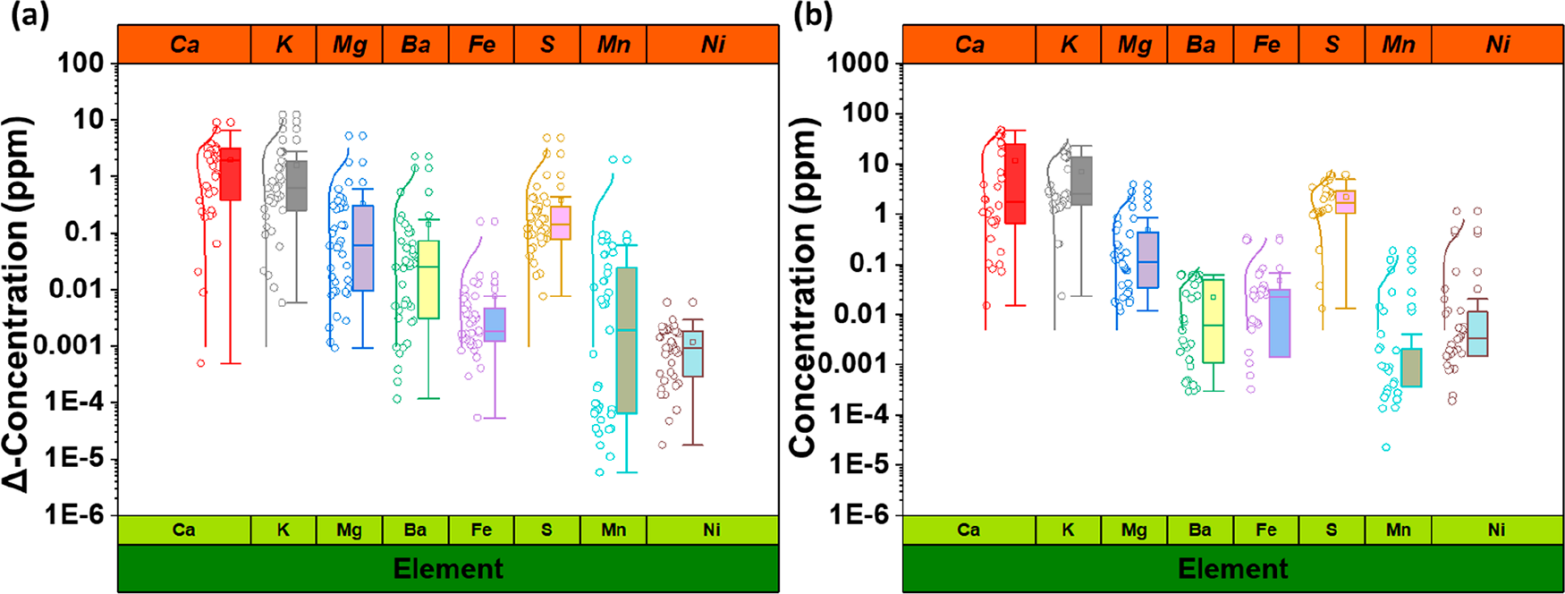
Sample fluid composition as determined by ICP-OES. (a) Differences in the concentration of a range of elements detected within fluid samples after completing the experiments. Each point belongs to two different experiments at strictly controlled identical conditions (repeated experiments). Plots in this panel contain 40 data points. (b) Differences in the concentration of a range of elements detected within fluid samples after completing the experiments without the presence of any sandstone (i.e., only brine in the experimental container with H_2_, N_2_, or no gas). Each point belongs to two different experiments at strictly controlled identical conditions (repeated experiments). Plots in this panel contain 32 data points. Refer to Figure 1 for the explanation of boxes, whiskers, and curved lines.

An important outcome of the experiments was the necessity to control each influencing parameter, either during preparation or during the running of the experiments. The chemistry of the brine, pressure, temperature, rock particle size, run-time, container type, and water-to-rock ratio, in addition to the presence of hydrogen, all have a bearing on the control of either chemical equilibria or reaction kinetics or both. This study is the first to observe and evaluate the effect of all these influencing parameters to ensure the highest-quality control over the experiments and the results. Most of the experiments with hydrogen in this study were conducted with particle sizes of less than 355 μm to accelerate the kinetics of any potential reaction. This was due to the observation that the use of larger rock size fractions was associated with no notable change in fluid composition (see Figure S6 and the Supporting Information data file). From the observed absence of geochemical reactions over a two-month period for those experiments conducted with smaller particle sizes, it can be concluded that no reactions would be observed for whole-rock samples over a longer time period (see Figure S7). The immediate implications from these results suggest that there is no risk of hydrogen loss and no risk for mineralogical and structural changes due to geochemical reactions in the investigated sandstone types, covering a wide range of mineralogies, and that the effect of any additional influencing parameters for each storage site, such as the presence of other gases or minerals, must be investigated over time scales of seasonal hydrogen storage before its usage. Moreover, hydrogen reactivity with cement must be understood to ensure that hydrogen will not degrade the wellbore cement over time, thereby preserving the integrity of the wellbore.

While the field of geological hydrogen storage in porous reservoirs is in its infancy, we believe it is essential to enhance the technological process at a conceptual level to help develop a real-world viability of this method and identify the parameters that will help make future field applications successful. The presented study was essential to reduce the uncertainties around the risk of hydrogen loss and reservoir integrity degradation due to geochemical reactions and establish the feasibility of large-scale hydrogen storage, paving the way for a new low-carbon energy storage technology that can support a major reduction in our carbon emissions. The experiments and analyses in this study have enabled us to solve one of the main scientific challenges and thereby reduce the risk associated with the geological storage of hydrogen, which is necessary to transition to a hydrogen economy and achieve net-zero emissions. In essence, considering the presented extensive experimental study, we conclude hydrogen storage in sandstone reservoirs is safe from the geochemical point of view and there is no expected hydrogen loss because of geochemical reactions.

## Experimental Methods

### Materials and Experimental Apparatus

Research-grade hydrogen (H_2_) and nitrogen (N_2_) gases (purity 99.9995 vol %) and sodium chloride (NaCl) of certified purity (99.5%) were supplied by BOC Ltd. and Fisher Scientific, respectively. Deionized water generated by an integral water purification system (ELGA DV 25) was used exclusively throughout the experiments. A range of sandstone samples were selected to capture an array of reservoir lithologies including two red aeolian Permian sandstones (Sand No. 1 and No. 2), the aeolian Hopeman sandstone (Sand No. 3), a shallow marine Carboniferous sandstone (Sand No. 4), and six aeolian Leman sandstone samples from the U.K. North Sea Rough Field (Sand No. 5).

**Figure 3:**
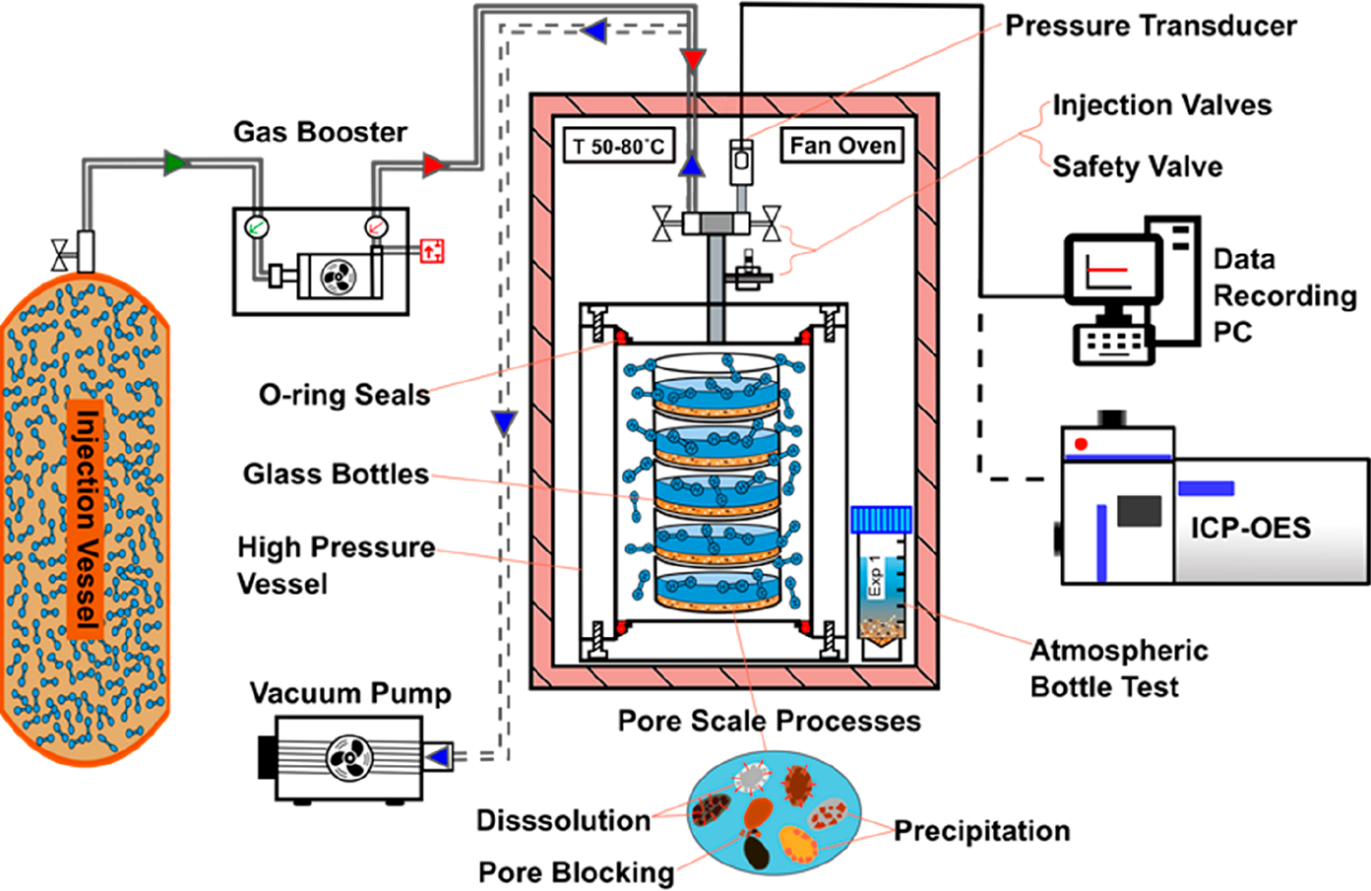
Experimental apparatus. Schematic diagram of high-pressure, static batch reactor, and bottle test experimental setup. Stepwise experimental procedure: vacuum extraction, gas pressurization and injection, mineral reaction processes, pressure and temperature monitoring, and ICP-OES analysis.

A fan oven (SciQuip Oven-110S) housed a series of 8 identical stainless steel high-pressure/temperature reactor vessels (volume 706 mL) containing glass sample bottles and atmospherically sealed sterile centrifuge tube containers (from Scientific Laboratory Supplies) made of medical grade polypropylene (bottle test) (Figure 3).

After the vessels were evacuated using a CPS VP2S pro-set single-stage vacuum pump, H_2_ and N_2_ gas were injected through a high-pressure valve at the top of the vessel. A Haskel air driven gas booster model 86980 (AG-75) was used to increase the gas pressure within the vessels. Vessel pressure and temperature conditions were measured continuously using a GD4200-US Digital Pressure Transducer from Elemental Science Inc. Data were recorded on a PC with LabVIEW software from National Instruments at 1 min intervals; the measurement errors for pressure and temperature were quantified as < ±0.15% span best fit straight line and ±1.5%FS total band, respectively.

### Experimental Design and Strategy

Bespoke static batch reactor experiments were designed and constructed to study the geochemical response of sandstones on exposure to hydrogen under in situ reservoir conditions. To closely recreate subsurface reservoir conditions and to ensure representative and consistent experimental methods and the repeatability of results, we controlled the experimental parameters of rock type, particle size, rock–water ratio, solution salinity, oxygen availability, temperature, and pressure (see Figure S8 and Tables S1 and S2).

Samples were disaggregated to grain sizes ranging between 0.335 and 4 mm to account for the role of (available) mineral surface area as a rate-controlling step in geochemical reactions.^27^ In lithified sediments, disaggregation reveals fresh mineral surfaces, promoting the occurrence of geochemical reactions which are representative of natural reservoir systems and are observable over laboratory time scales.^28^ Some of the samples were sterilized (heated at 120 °C for 1 h) to remove micro-organisms that could have promoted unwanted biologically induced hydrogen reactions.^29^

Aqueous solutions of salinities 0, 35, 100, and 250 parts per thousand (ppt) NaCl were used. Deionized water was utilized as a control for gas–water–rock chemical reactions associated with the reaction vessel. The analysis of 0 ppt NaCl solutions ensures that the effect of salinity-associated salting-out effects in nonpolar molecules does not result in the solubility of H_2_ decreasing and preventing associated geochemical reactions from occurring.^31^ A set of experiments were undertaken with different rock–water ratios to evaluate the rate-dependent effect of the mineral phase concentration on hydrogen-associated geochemical reactions. The experiments utilized glass bottles and centrifuge tube containers rather than stainless steel to prevent contamination from steel corrosion and degradation.^32^

In preparation for gas injection, free oxygen (O_2_) in each vessel was removed by vacuum degassing^33^ and nitrogen flow through for 1 h.^34^ O_2_ removal ensured experimental conditions replicated an anoxic environment. Following sample anoxification, batch reactor vessels were injected with either H_2_ or N_2_ gas to pressures ranging from 1 to 20 MPa. Experimental controls were conducted with inert nitrogen (control experiments) to ensure that any observed geochemical reactions were induced by the presence of hydrogen. Bottle tests (control experiments) with no injected gas were conducted for each sample in sealed atmospheric-pressure containers to quantify any pressure dependence of the hydrogen–sandstone reactivity.

An experimental matrix was designed to ensure each variable within the geochemistry experiments (hydrogen, temperature, pressure, salinity, and rock type) could be independently evaluated. The batch reaction experiments were conducted at temperatures ranging from 332.15 up to 353.15 K, representative of probable storage reservoir conditions. Precise temperature regulation and monitoring (±0.1 K) with samples held within an oven throughout the experimental period limited the influence of temperature fluctuations on any geochemical reactions. The experimental duration ranged from 2 to 8 weeks, encapsulating the role of reaction kinetics in hydrogen- sandstone reactions. The complete experimental details and matrix are provided in the Supporting Information.

### Measurements and Analysis

**Figure 4:**
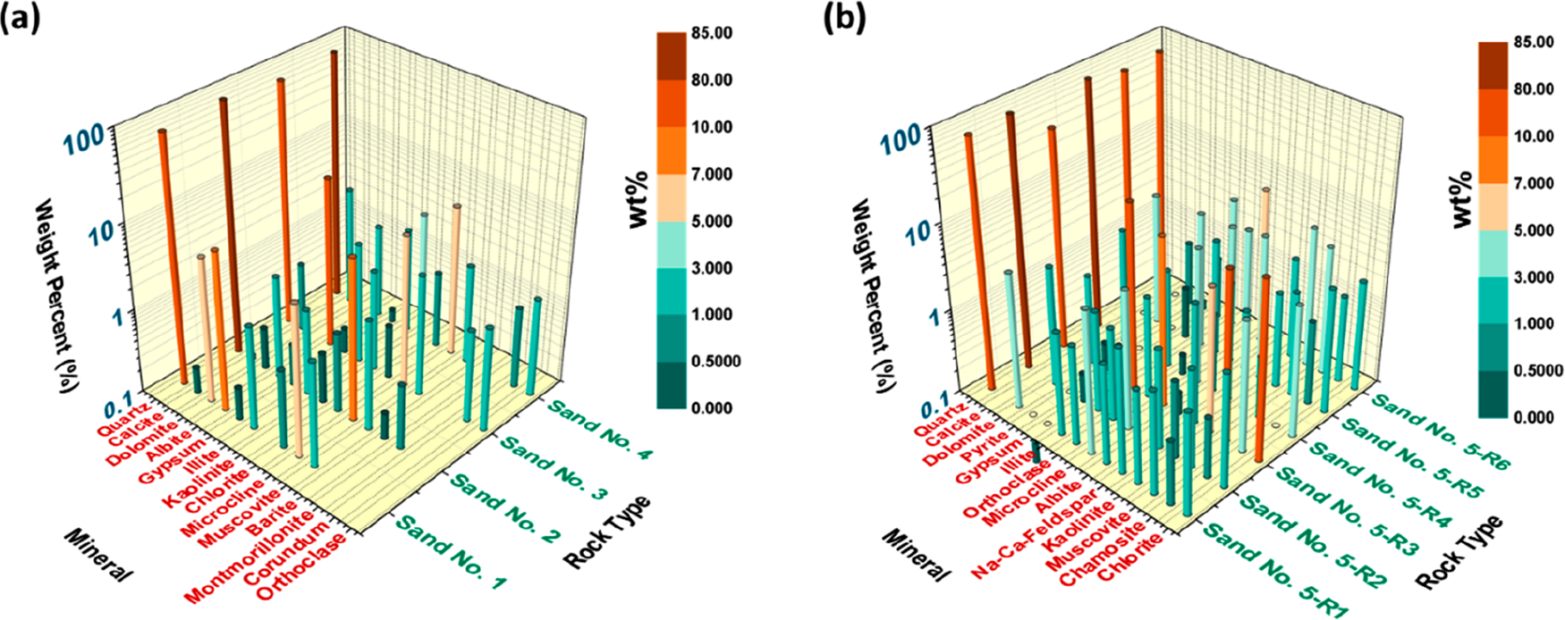
Mineral composition of sandstones. Bulk mineral composition of each sample as determined by XRD analysis; comparison to samples shows compositional variability (see Tables S3 and S4).

Sample mineralogy (see Figure 4) was determined by X-ray diffraction (Bruker D8 - Powder Diffractometer: scanning parameters 0–90°, 2θ, accuracy in peak positions ≤0.01 2θ, Bragg–Brentano configuration). Mineral phases were identified using the internal Bruker database with EVA analysis package, and weight percentages (wt %) were quantified by Rietveld analysis. The sample fluid composition was determined both preceding and after batch reactor experiments by inductively coupled plasma–optical emission spectroscopy (ICP-OES) using a Varian Vista Pro with APEX-E from Elemental Science Inc. (LoD of 0.105 × 10^3^ to 0.26 ppm or ∼ 0.2–100 ppb (see Figure S1)). The related data can be found in the Supporting Information data file. A Hiden HPR-20 triple filter mass spectrometer with an ultimate detection limit of 5 ppb was used to measure the concentration of the reacted gas after completing the experiments. Further analysis details can be found in the Supporting Information.

### Safety Measures

The safe operation of the high-pressure/temperature batch reaction vessels was ensured by appropriate experimental design and engineering. The use of 316 stainless steel (high Mn, <13% Ni) reduced the susceptibility of reaction vessels to degradation and blistering.^32,35^ Vessel tops were secured by 8 M12 × 35 mm high tensile cap screws and O-ring seals yielding upper limits of 65 MPa. High-pressure valves and instruments sourced from Top Industrie were used throughout the experiments, ensuring tolerance and reliability under extreme conditions (up to 100 MPa). During hydrogen injection, the rate was carefully regulated to prevent the potentially dangerous, rapid heating of vessels by the Joule–Thomson effect.^36^ A hydrogen gas alarm (Riken Keiki GD-A80 detector head with HW-6211 sensor and GP-6001 single-channel monitor panel) was fitted in the lab as an additional safety measure in case of leakages.

## Supporting Information Available

ICP-OES limit of detection (LoD), complementary fluid compositions, composition of gas phase after completing the experiments, recorded pressure and temperature of experiments, effect of particle size on mineral dissolution, concentrations of elements with time, and experimental details (PDF)
Detailed experimental data (XLSX)
