# Supplementary material for: Geological Hydrogen Storage: Geochemical Reactivity of Hydrogen with Sandstone Reservoirs
Source: ACS Energy Lett. 2022 Jun 3;7(7):2203–10. doi: 10.1021/acsenergylett.2c01024 (PMC9274762; doi:10.1021/acsenergylett.2c01024)
Supplement: Supplementary file 1 — nz2c01024_si_001.pdf [file nz2c01024_si_001.pdf]

SUPPORTING INFORMATION

Geological hydrogen storage: Geochemical reactivity of hydrogen with sandstone reservoirs

Aliakbar Hassanpouryouzband[1*], Kate Adie[1], Trystan Cowen[1], Eike M Thaysen[1], Niklas Heinemann[1], Ian B. Butler[1], Mark Wilkinson[1], Katriona Edlmann[1*]

[1]School of Geosciences, University of Edinburgh, Grant Institute, West Main Road, Edinburgh, EH9 3FE, UK

[*] Corresponding authors: A.H: Hssnpr@ed.ac.uk, K.E: katriona.edlmann@ed.ac.uk

Supporting Figures

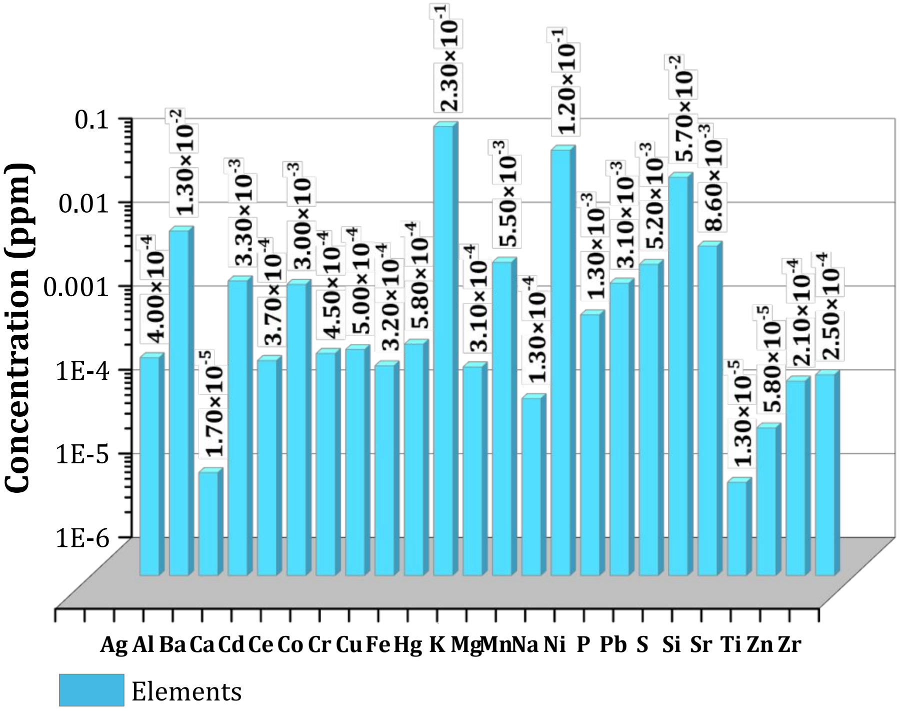

Fig. S1 ICP-OES limit of detection (LoD): The lowest concentration of the elements that can be reliably detected and quantified using the ICP-OES within this study.

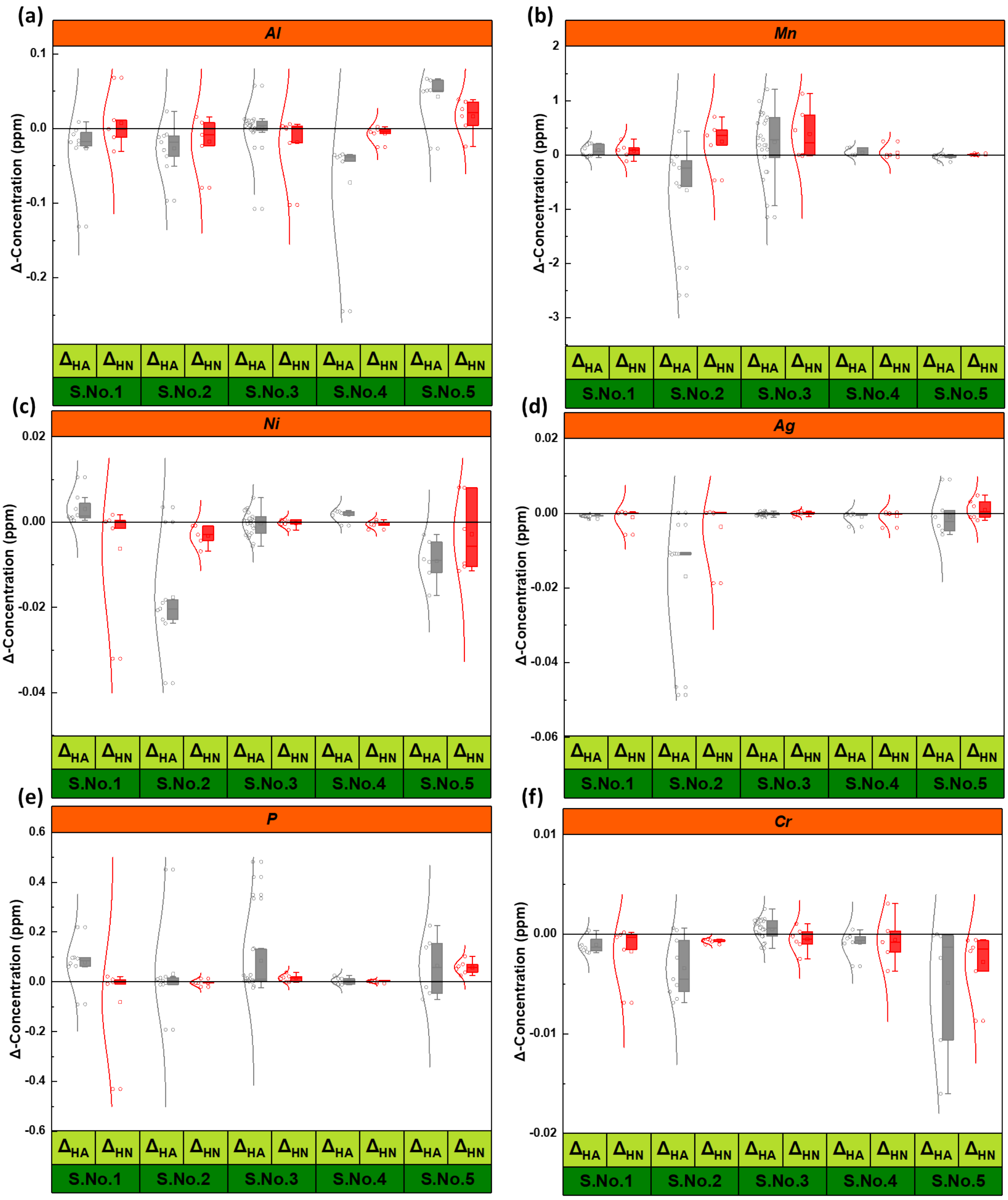

Fig. S2 Differences in the concentrations of different elements detected in fluid samples after completing the experiments. Each point belongs to two different experiments at strictly controlled identical conditions with only one changing parameter that is presence of H_2 or N_2, or a lack of any gas phase. ΔHN: concentration of the element after experiment with H_2 deducted by the concentration of the same element after experiments with N_2. ΔHA: concentration of the element after experiment with H_2 deducted by the concentration of the same element after the bottle test. The boxes are determined by the 25th and 75th percentiles and the whiskers are extended to a maximum of 1.5 × IQR beyond the boxes. The curved lines represent the distribution curves. Each plot contains the following number of data points: (S.No.1-ΔHA:8), (S.No.1-ΔHN:5), (S.No.2-ΔHA:9), (S.No.2-ΔHN:5), (S.No.3-ΔHA:23, (S.No.3-ΔHN:6), (S.No.4-ΔHA:6, (S.No.4-ΔHN:5), (S.No.5-ΔHA:6, (S.No.5-ΔHN:6). Detailed data about the concentration of elements can be found in the supporting data file.

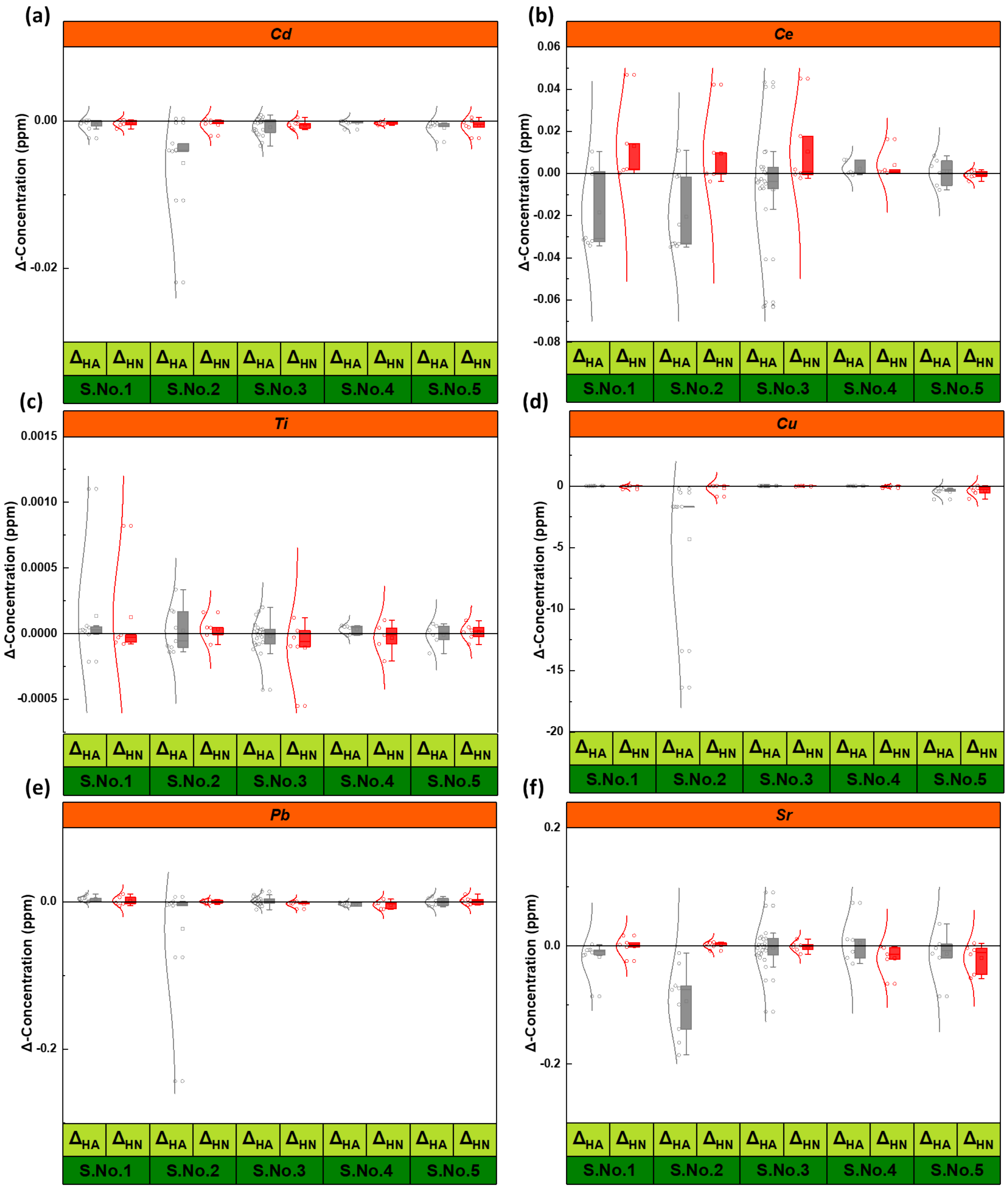

Fig. S3 Differences in the concentrations of different elements detected in fluid samples after completing the experiments. Each point belongs to two different experiments at strictly controlled identical conditions with only one changing parameter that is presence of H_2 or N_2, or a lack of any gas phase. ΔHN: concentration of the element after experiment with H_2 deducted by the concentration of the same element after experiments with N_2. ΔHA: concentration of the element after experiment with H_2 deducted by the concentration of the same element after the bottle test. The boxes are determined by the 25th and 75th percentiles and the whiskers are extended to a maximum of 1.5 × IQR beyond the boxes. The curved lines represent the distribution curves. Each plot contains the following number of data points: (S.No.1-ΔHA:8), (S.No.1-ΔHN:5), (S.No.2-ΔHA:9), (S.No.2-ΔHN:5), (S.No.3-ΔHA:23, (S.No.3-ΔHN:6), (S.No.4-ΔHA:6, (S.No.4-ΔHN:5), (S.No.5-ΔHA:6, (S.No.5-ΔHN:6). Detailed data about the concentration of elements can be found in the supporting data file.

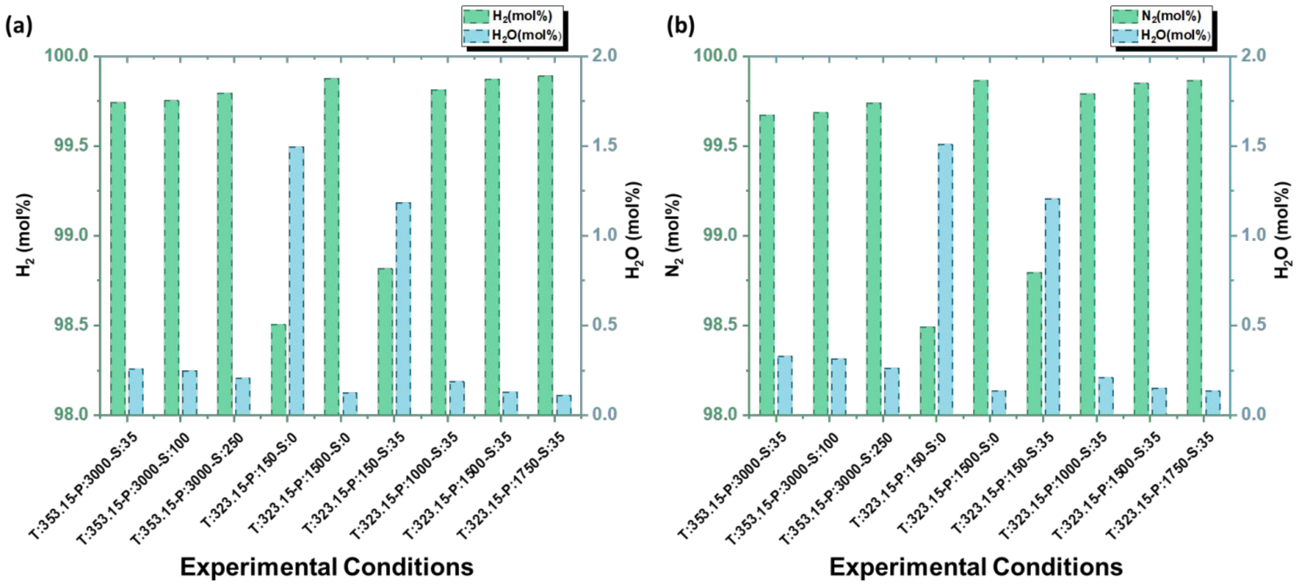

Fig. S4 Composition of gas phase after completing the experiments in presence of H_2(a) and N_2(b). The only observed impurity within the gas phase was water vapour. We observed that the range of water fractions within the gas phase follows the thermodynamics rules; decreasing with pressure and salinity and increasing with temperature. There were no other gases detected in the gas composition of any of the experiments at any concentration above ppb levels (lowest detection limit of the mass spectrometry device). In the X-axis, T shows temperature in K, P shows pressure in psi, and S indicates salinity in ppt.

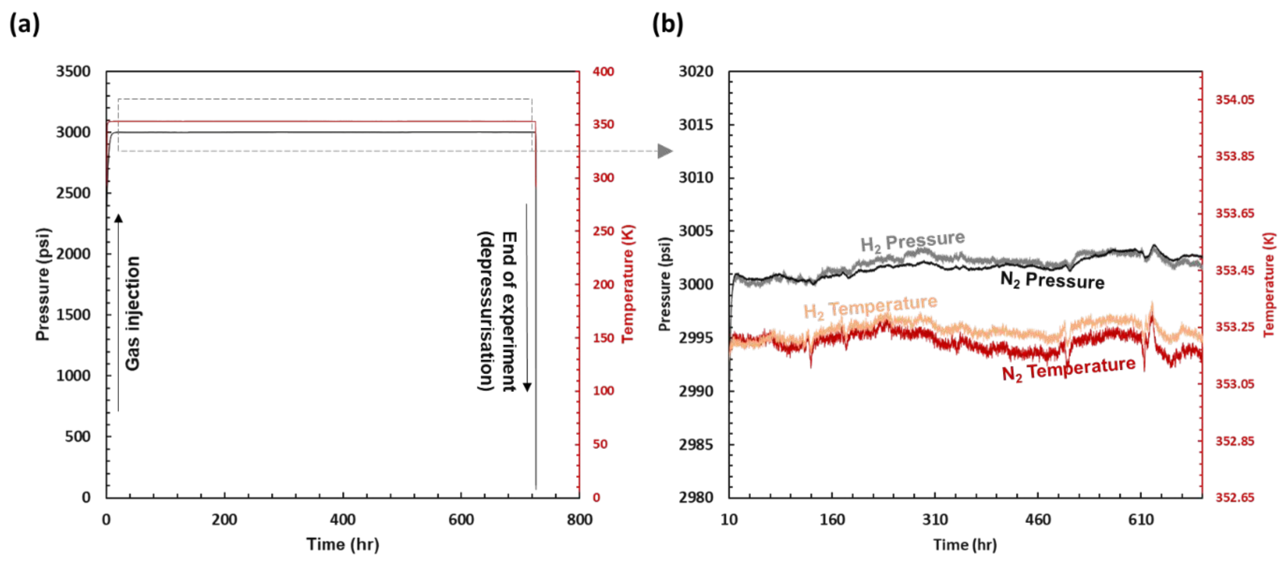

Fig. S5 (a) The recorded pressure and temperature of the batch reaction vessels during the experiments, magnified in (b) for the entire duration of the experiment after gas injection. A precise monitoring system was in place to measure the pressure and temperature of the system during experiments. As can be seen from (a), no notable changes in pressure and temperature were observed. The very small temperature changes occurring inside the oven are responsible for the fluctuations observed in (b) that are so small they can be deemed negligible.

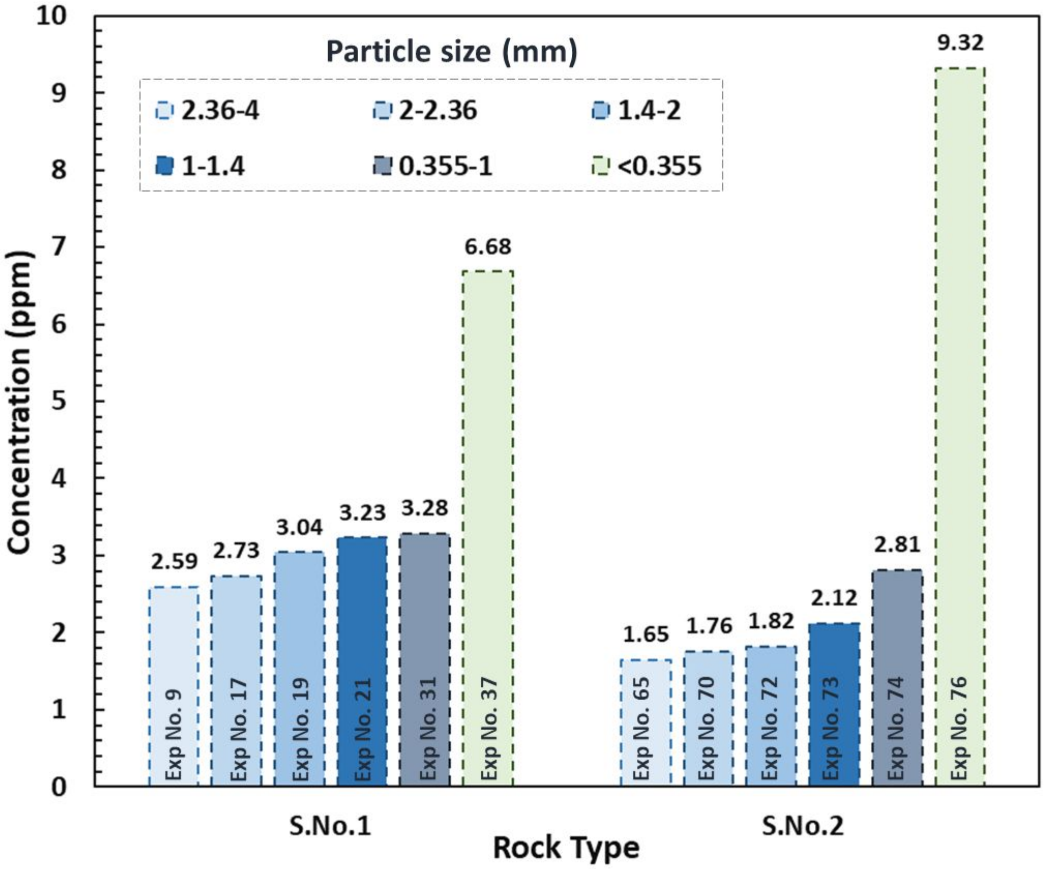

Fig. S6 Concentrations of Si elements in the fluid samples after completing the experiments for two different types of sandstone rocks with different particle sizes. Except for the particle size, all other experimental conditions were identical for each sandstone tested. The experimental conditions were as follows: Temperature: 323.15 K, Pressure: Ambient with no gas, Salinity: 35ppt-brine, brine amount: 50gr, Rock sample amount: 15gr, Run Time: 2 weeks. Experimental numbers in provided supporting data file for each experiments are as follows: S.No.1: 9(2.36-4), 17(2-2.36), 19(1.4-2), 21(1-1.4), 31(0.355-1), 37 (<0.355); S.No.2: 65(2.36-4), 70(2-2.36), 72(1.4-2), 73(1-1.4), 74(0.355-1), 76(<0.355). The data clearly shows that higher reaction rates are associated with smaller rock sizes.

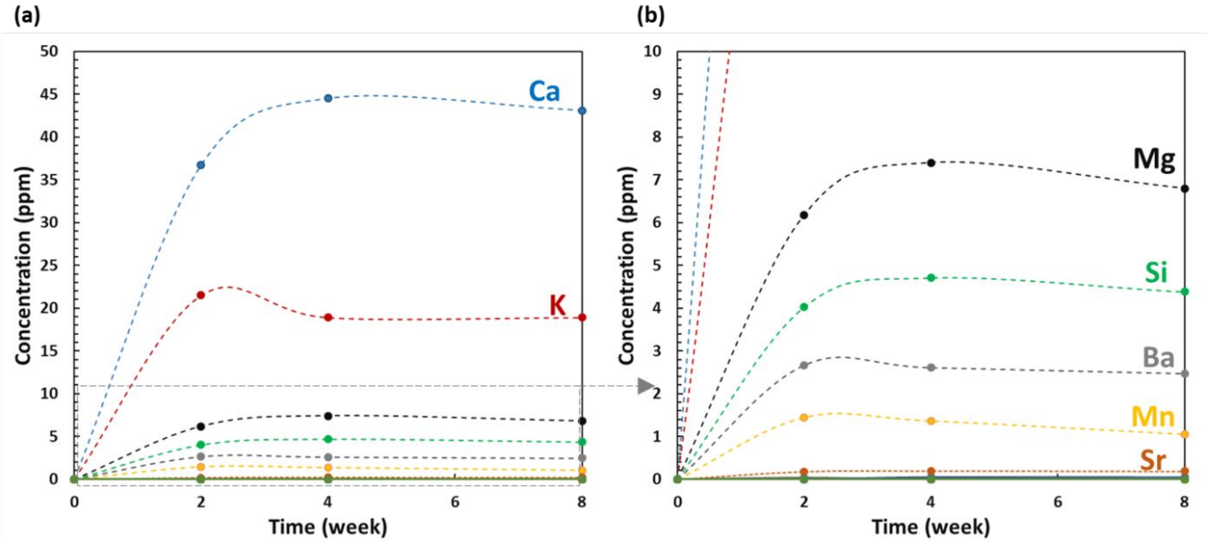

Fig. S7 Changes in the concentrations of different elements within fluid samples after completing the experiments in the presence of hydrogen, with the lower concentrations magnified (b). Except for the reaction times, all other experimental conditions were identical. The experimental conditions are as follows: Rock Type: S.No.3: Temperature: 323.15 K, Pressure: 150psi, Salinity: 35ppt, Particle diameter: <0.355 mm, brine amount: 50gr, Rock sample amount: 15gr. Experimental numbers in provided supporting data file for each experiments are as follow: 118 (2 weeks), 126 (1 Month), 132 (2 Months). As can be seen, the disaggregation of rock samples to smaller particles created a higher reaction rate and a more stable fluid composition is achieved over a shorter time span. The dotted lines are for clarity and do not reflect a best fit to the data

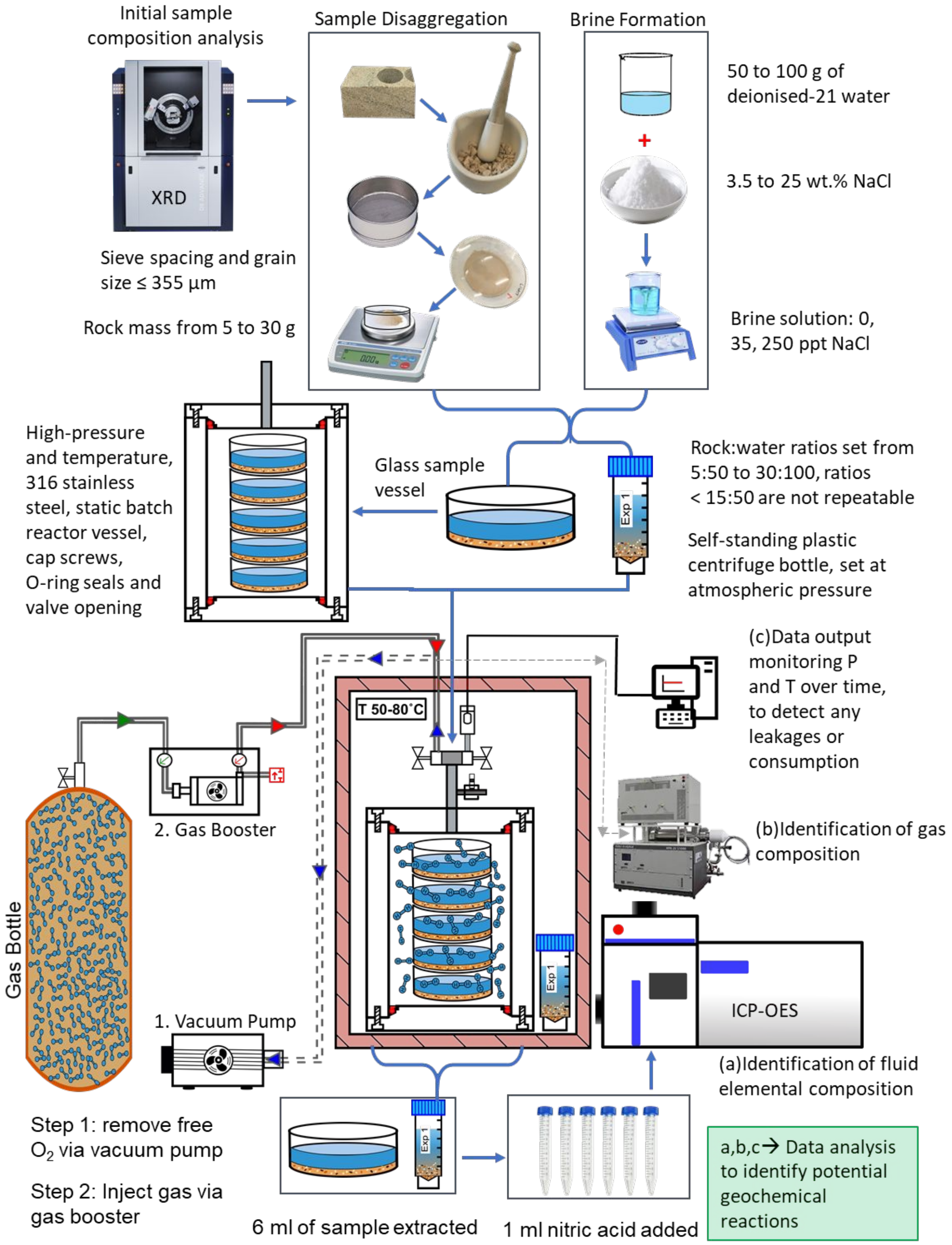

Fig. S8 The step by step process of setting up the batch reaction experiments and analysing the data is illustrated graphically. The figure summarises the whole experimental process, beginning with characterising the rock samples with XRD through preparing the fluid, onto batch reaction setup and finishing with the data analysis based on the methodology described.

Supporting Tables

Table S1. Supplementary Methodology: List of experimental parameters used in this study. High-pressure batch reaction experiments were used to accurately study the geochemical interactions between gases, fluids, and materials under a range of subsurface reservoir conditions.

Experimental parameters and ranges		
**Parameter**	**Unit**	**Range**
Temperature	Kelvin (K)	332.15 - 353.15 K
Pressure	Mega Pascal (MPa)	1 - 20 MPa
Salinity	Parts per trillion (ppt)	0 - 250 ppt
Grain Size	millimeter (mm)	<0.355 - 4 mm
Rock Mass	Grams (g)	0 - 30 g
Water/brine Mass	Grams (g)	50 - 100 g
Run Time	Weeks	1 - 8 weeks
Gas Type	Composition	No gas(ambient), N_2, H_2
Vessel Type	Composition	glass, polypropylene, steel

Table S2. Experimental controls: Summary of the controls and criteria that were assessed to ensure the experimental results were repeatable and robust.

Experimental controls and repeatability	
Controls	An extensive matrix of experimental controls ensured repeatability, which include; pressure (1 to 20 MPa), temperature (50 to 80 C°), salinity (0 to 250 ppt), vessel conditions, gas compositions (oxygen, nitrogen, and hydrogen), grain sizes and rock to water ratios (15:50)
Temperature	Experiments undertaken in an thermostatically controlled oven where temperatures up to 80°C are reliable and repeatable due to precise temperature regulation and monitoring which limited any potential temperature-dependent effects on potential geochemical reactions.
Pressure	Bottle tests undertaken in sealed atmospheric plastic containers with no injected gas accounted for any pressure-dependent effects on mineral reactions in each experiment. Therefore, experiments at pressures between 1 - 20 MPa were validated and deemed repeatable.
Salinity	Experiments at salinities up to 250 ppt are reliable and repeatable.
Sample sterilization	No effect on the results were observed due to sample sterilisation. Sterilisation is an essential preparation step for the rock samples to minimise the potential occurrence of biotic reactions that may influence component concentrations, particularly the gas composition.
Rock to water ratio	Each sample was examined at different rock-water ratios to evaluate the rate-dependent effect of mineral phase concentration on hydrogen associated geochemical reactions.
Grain size	Grain sizes from 0.335 to 4 mm are suitable however, larger grain sizes must be balanced by a higher rock to water ratio. Smaller grain sizes increase mineral reactive surface areas resulting in higher concentrations of dissolved components. Uniform grain size across all experimental conditions for an individual rock type was implemented to ensure a more robust analysis of sample reactivity.
Reaction vessel	High-pressure/temperature, 316 stainless steel, static batch reactor with O-ring seals and high tensile cap screws. No degradation or blistering was observed on the steel or O-rings under this range of parameters.
Sample vessel	Tested with steel - contamination identified - changed to glass bottles, self-standing plastic centrifuge bottles used for atmospheric tests
Oxygen removal	Nitrogen flow through and vacuum pumping was utilised on all experiments to remove free oxygen from the vessels before gas injection, controlling the redox-sensitive nature of anoxic subsurface environments.

Repeatability	Overall experimental repeatability is high with robust results produced across 253 experiments including 40 repeats covering all conditions. Replacement of batch reactor O-ring seals after each experimental cycle.

Table S3. Rock composition: Bulk mineral composition of sample S No.1-4 as determined by XRD analysis, comparison to highlight any compositional variability between samples. The selected samples originated from localised locations within their respective geological formations, and as such the small volume of rock analysed will not provide a representative elementary volume (REV) of the entire geological formation, but they are representative of the formation being investigated. The data quality implications of sampling bias and REVs from the XRD compositional data was accounted for by conducting three repeat XRD analysis per sample of each rock type, to identify any internal variability within the sandstone types tested and bulk mineralogy was obtained by taking an average wt.% for each mineral type.

Sand No. / Mineral (wt%)	S No.1	S No.2	S No.3	S No.4
Quartz	76.63	84.56	72.43	80.43
Calcite	0.2	0.11	0.66	2.48
Dolomite	5.18	0.31	0	0
Albite	7.54	1.66	10.31	1.29
Gypsum	0.24	0.31	0.2	0.14
Illite	1.64	1.03	2.65	1.74
Kaolinite	0	0.39	1.58	3.26
Chlorite	0.83	0.87	0.42	0.8
Microcline	6.03	8.04	6.21	6.01
Muscovite	1.71	1.95	0	0
Barite	0	0.2	0	0
Montmorillonite	0	0.57	0	0
Corundum	0	0	1.2	0.9
Orthoclase	0	0	1.63	1.45
Muscovite	0	0	2.71	1.5

Table S4. Rock composition: Bulk mineral composition of Sample No.5 (subsamples R1-R6), which represent six different samples from the Rough field, as determined by XRD analysis. Comparisons between the different samples show that there is compositional variability.

Sand No. / Mineral (wt%)	S No. 5-R1	S No. 5-R2	S No. 5-R3	S No. 5-R4	S No. 5-R5	S No. 5-R6
Quartz	78.75	83.6	38	82.779	68.392	74.06
Calcite	0	0	0	0	0.012	0.01
Dolomite	4	2.65	1.19	2.4	3.71	0.58
Pyrite	0.03	0.02	0.22	0.051	0.046	0.12
Gypsum	0.08	0.22	0.26	0	0.42	0.56
illite	1.63	1.55	15.3	1.5	4.05	3.54
Orthoclase	1.46	1.24	1.6	0.17	2.39	1.93
Microcline	4.71	4.36	9.65	4.13	4.23	6.76
Albite	1.48	0	0.18	0.86	4.74	0
Na-Ca-Feldspar	2.89	1.47	2.7	2.53	4.97	1.58
Kaolinite	1.26	0.81	5.2	1.48	1.36	4.46
Muscovite	1.59	1.42	10	0.54	1.73	3.31
Chamosite	0.56	0.51	3.4	0.09	0.97	1.08
Chlorite	1.56	2.15	12.3	3.47	2.98	2.01

Supporting Data

Excel file: Details of experimental conditions and the results of ICP-OES after completing the experiments are provided in the supporting excel file. Each row belongs to one experiment.
